# Supplementary material for: A fungal pathogen induces systemic susceptibility and systemic shifts in wheat metabolome and microbiome composition
Source: Nat Commun. 2020 Apr 20;11:1910. doi: 10.1038/s41467-020-15633-x (PMC7171108; doi:10.1038/s41467-020-15633-x)
Supplement: Supplementary file 3 — Description of Additional Supplementary Files [file 41467_2020_15633_MOESM3_ESM.pdf]

Description of Additional Supplementary Files

File Name: Supplementary Data 1
Description: Annotation list of plant secondary metabolites identified in Obelisk and Chinese Spring during the metabolomics study.

File Name: Supplementary Data 2
Description: List of differentially accumulating metabolites with significant differences in at least one of the three comparisons. Number of biologically independent replicates: n=3. For details on the statistical analysis of the metabolomics dataset, please see the methods section. *P
