# Supplementary material for: A fungal pathogen induces systemic susceptibility and systemic shifts in wheat metabolome and microbiome composition
Source: Nat Commun. 2020 Apr 20;11:1910. doi: 10.1038/s41467-020-15633-x (PMC7171108; doi:10.1038/s41467-020-15633-x)
Supplement: Supplementary file 6 — Reporting Summary [file 41467_2020_15633_MOESM6_ESM.pdf]

nature research

Corresponding author(s): estukenbrock@bot.uni-kiel.de

Last updated by author(s): Mar 10, 2020

Reporting Summary

Nature Research wishes to improve the reproducibility of the work that we publish. This form provides structure for consistency and transparency in reporting. For further information on Nature Research policies, see Authors & Referees and the Editorial Policy Checklist .

Statistics

For all statistical analyses, confirm that the following items are present in the figure legend, table legend, main text, or Methods section.

n/a	Confirmed	
☐	☒	The exact sample size (n) for each experimental group/condition, given as a discrete number and unit of measurement
☐	☒	A statement on whether measurements were taken from distinct samples or whether the same sample was measured repeatedly
☐	☒	The statistical test(s) used AND whether they are one- or two-sided<br>*Only common tests should be described solely by name; describe more complex techniques in the Methods section.*
☐	☒	A description of all covariates tested
☐	☒	A description of any assumptions or corrections, such as tests of normality and adjustment for multiple comparisons
☐	☒	A full description of the statistical parameters including central tendency (e.g. means) or other basic estimates (e.g. regression coefficient) AND variation (e.g. standard deviation) or associated estimates of uncertainty (e.g. confidence intervals)
☐	☒	For null hypothesis testing, the test statistic (e.g. F, t, r) with confidence intervals, effect sizes, degrees of freedom and P value noted<br>*Give P values as exact values whenever suitable.*
☒	☐	For Bayesian analysis, information on the choice of priors and Markov chain Monte Carlo settings
☒	☐	For hierarchical and complex designs, identification of the appropriate level for tests and full reporting of outcomes
☐	☒	Estimates of effect sizes (e.g. Cohen's d, Pearson's r), indicating how they were calculated

Our web collection on statistics for biologists contains articles on many of the points above.

Software and code

Policy information about availability of computer code

Data collection	ImageJ was used for data extraction from scanned infected wheat leaves.
Data analysis	R (3.4.2)<br>All R scripts used for the microbiome analyses are accessible through GitHub https://github.com/hmamine/ZIHJE.<br>Qiime2 https://qiime2.org/<br>BIOM Format http://biom-format.org<br>Metabolome anaysis-specific software from Bruker: DataAnalysis (5.0), MetaboScape (4.0.1)

For manuscripts utilizing custom algorithms or software that are central to the research but not yet described in published literature, software must be made available to editors/reviewers. We strongly encourage code deposition in a community repository (e.g. GitHub). See the Nature Research guidelines for submitting code & software for further information.

Data

Policy information about availability of data

All manuscripts must include a data availability statement. This statement should provide the following information, where applicable:

- Accession codes, unique identifiers, or web links for publicly available datasets
- A list of figures that have associated raw data
- A description of any restrictions on data availability

A reporting summary for this Article is available as a Supplementary Information file. The source data underlying Figs. 1, 2 and 6 as well as Supplementary Figs. 4, 5, 6 and 10 are provided as a Source Data file. Raw data from microbiota profiling is available in the NCBI BioProject database under https://www.ncbi.nlm.nih.gov/bioproject/PRJNA549447. Data generated and analysed during the metabolome study are available from the corresponding author upon request.

Field-specific reporting

Please select the one below that is the best fit for your research. If you are not sure, read the appropriate sections before making your selection.

☒ Life sciences ☐ Behavioural & social sciences ☐ Ecological, evolutionary & environmental sciences

For a reference copy of the document with all sections, see nature.com/documents/nr-reporting-summary-flat.pdf

Life sciences study design

All studies must disclose on these points even when the disclosure is negative.

Sample size	Selection of sample size was based on a combination of extensive experience with respect to the variation inherent to each of the experimental systems described in the manuscript as well as assurance of rigorous handling of all samples. The respective sample size is stated for every result shown in the manuscript.
Data exclusions	No data were excluded from any analysis except for variables as described in "Statistical analysis of metabolomics dataset" in the Supplementary Methods section.
Replication	The respective number of replications of each experiment is stated in the manuscript. The metabolome anaysis as well as the microbiome study were conducted once. No replication was performed that was not documented in the paper.
Randomization	In all experiments, plants for the respective experimental groups were selected randomly and harvested in a random order.
Blinding	For metabolomics measurements blinded samples identifiers were used. Unblinding of samples took place after primary data quality control.

Reporting for specific materials, systems and methods

We require information from authors about some types of materials, experimental systems and methods used in many studies. Here, indicate whether each material, system or method listed is relevant to your study. If you are not sure if a list item applies to your research, read the appropriate section before selecting a response.

Materials & experimental systems

n/a	Involved in the study	
☒	☐	Antibodies
☒	☐	Eukaryotic cell lines
☒	☐	Palaeontology
☒	☐	Animals and other organisms
☒	☐	Human research participants
☒	☐	Clinical data

Methods

n/a	Involved in the study	
☒	☐	ChIP-seq
☒	☐	Flow cytometry
☒	☐	MRI-based neuroimaging
